# Effects of Sintering Conditions on Structures and Properties of Sintered Tungsten Heavy Alloy

**DOI:** 10.3390/ma13102338

**Published:** 2020-05-19

**Authors:** Lenka Kunčická, Radim Kocich, Zuzana Klečková

**Affiliations:** 1 Institute of Physics of Materials, CAS, 61662 Brno, Czech Republic; 2 Faculty of Materials Science and Technology, VŠB-Technical University of Ostrava, 70833 Ostrava 8, Czech Republic; radim.kocich@vsb.cz (R.K.); zuzana.kleckova@vsb.cz (Z.K.)

**Keywords:** tungsten heavy alloy, powder metallurgy, sintering, quenching, microstructure

## Abstract

Probably the most advantageous fabrication technology of tungsten heavy alloys enabling the achievement of required performance combines methods of powder metallurgy and processing by intensive plastic deformation. Since the selected processing conditions applied for each individual processing step affect the final structures and properties of the alloys, their optimization is of the utmost importance. This study deals with thorough investigations of the effects of sintering temperature, sintering time, and subsequent quenching in water on the structures and mechanical properties of a 93W6Ni1Co tungsten heavy alloy. The results showed that sintering at temperatures of or above 1525 °C leads to formation of structures featuring W agglomerates surrounded by the NiCo matrix. The sintering time has non-negligible effects on the microhardness of the sintered samples as it affects the diffusion and structure softening phenomena. Implementation of quenching to the processing technology results in excellent plasticity of the green sintered and quenched pieces of almost 20%, while maintaining the strength of more than 1000 MPa.

## 1. Introduction

Given by the requirements on high density, high strength, and favorable toughness and ductility, tungsten heavy alloys (THAs) are advantageously used for demanding applications, such as for radiation shielding, space industry components, therapeutic devices in oncology, aircraft counterbalances, or kinetic penetrators [1,2,3]. THAs can be fabricated via modern technologies, such as spark plasma sintering (SPS), or selective laser melting (SLM) [4,5,6,7,8]. However, the processing parameters, which can be varied during such procedures, are limited. Probably the most advantageous THA fabrication technology enabling the achievement of required performance of the final product combines methods of powder metallurgy and processing by intensive plastic deformation, which can preferably be performed by severe plastic deformation (SPD) methods [9,10,11,12,13,14] imparting significant grain refinement, down to the ultra-fine scale (0.1–1.0 µm, e.g., equal channel angular pressing—ECAP [15]), or even to nano-scale (≤100 nm, e.g., high pressure torsion—HPT [16]), resulting in the enhancement of mechanical and utility properties via imposing high shear strain. 

THAs typically consist of 90 to 97 wt. % of tungsten plus a mixture of other relatively low melting elements, such as Fe, Ni, Co, and Cu, the combination and volume fractions of which significantly influence the strength and plastic properties of the final product [17,18,19,20]. The tungsten content can be increased up to 98 wt. % to improve the strength, however, the strength usually increases at the expense of ductility; the content of matrix-forming elements lower than 3 wt. % usually supports brittleness of the final product [21]. On the other hand, increasing the addition of alloying, i.e., matrix-forming, elements generally decrease strength, but increase ductility [22]; high contents of matrix-forming elements contribute to uneven shape of the cross-section of the sintered piece due to gravity sedimentation during sintering, and consequently to non-uniformity of mechanical properties.

THAs are mostly consolidated from initial powder mixtures through powder metallurgy techniques; preferably via liquid phase sintering (LPS) [23], the boundaries of the grains during which melt and ensure binding of the tungsten particles and elimination of porosity via diffusion [24]. LPS offers the advantages of a relatively low processing temperature, favorable densification and structure homogenization, high productivity, and minimum production waste (<5%) [25]. Under optimized sintering conditions, the W and NiCo phases are homogenously distributed and the sintered piece contains no visible pores [7]. Sintering is typically performed at temperatures between 1000 and 1500 °C to ensure melting of the matrix-forming binding elements [22,26,27]. The green sintered THA work pieces typically feature high density (16–18 g·cm^−3^), and relatively high strength and plasticity. Nevertheless, besides the chemical composition, the absolute values of the mechanical properties after sintering depend on the character of the sintered structure, and, last but not least, also on the subsequent processing steps (the mechanical properties of THAs can also be enhanced by post-sintering deformation processing).

The presented work deals with the effects of sintering conditions, sintering temperature, and time in particular, and possible quenching on the structure and basic mechanical properties of the studied WNiCo tungsten heavy alloy. The primary goal is to optimize the sintering procedure in order to provide the best possible starting conditions for subsequent deformation processing.

## 2. Materials and Methods

**Figure 1:**
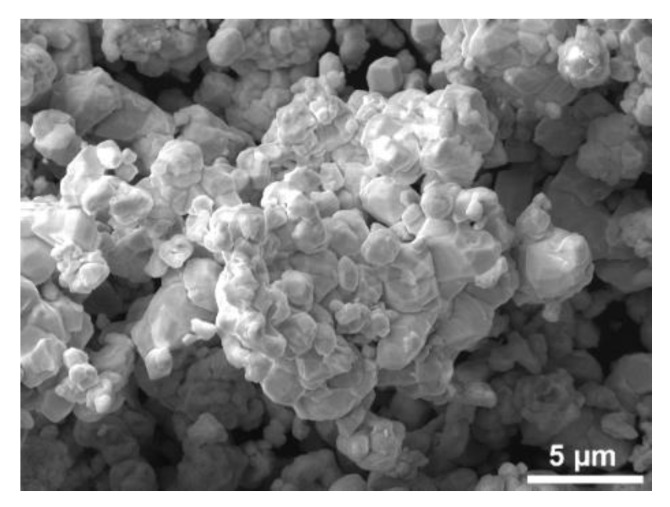
SEM image of original powder mixture.

The investigated 93W6Ni1Co tungsten heavy alloy samples were prepared at ÚJP Praha a.s. company (Praha, Czech Republic) from a homogeneous mixture of individual powders prepared by mechanical alloying (impurities ~<13 ppm of Fe, Cr, Mo, Al, and Ca). The granulometric distribution of the powder particles within the mixture was between 2 and 4 µm, and the mean particle size was 2.78 μm. The scanning electron image of the powder is depicted in Figure 1. After powders mixing, the mixture was cold isostatically pressed at 400 MPa, and subsequently prepared in the following steps:

(1) sintering under a protective atmosphere (hydrogen) at temperatures varying between 1450 and 1550 °C, with the step of 25 °C, for 30 min each (the temperature range was suggested by the ÚJP Praha a.s. company based on their previous experience);

(2) optional quenching in water-a set of samples was quenched after the mentioned sintering procedures in order to observe possible changes in structures and properties;

(3) based on the acquired data, additional experiments with variable sintering time were performed-sintering for 60 to 180 min, with the step of 30 min, at the sintering temperature of 1500 °C, and sintering for 180 min at the sintering temperature of 1525 °C.

The following structure analyses performed on cross-sectional cuts of the sintered (and possibly quenched) pieces primarily focused on structure character and possible changes in distribution of the individual elements within the structure. The transversally cut cross-sectional samples were ground mechanically and polished vibrationally using a colloidal silica suspension (VibroMet 2 Vibratory Polisher, Buehler, Esslingen, Germany) and subjected either to optical microscopy using an Olympus DSX1000 digital microscope (Olympus Czech Group, s.r.o., Prague, Czech Republic), or to scanning electron microscopy (SEM) using a Tescan Lyra 3 device (TESCAN Brno s.r.o, Brno, Czech Republic). TEM images were acquired on ion polished thin foils using a JEOL 2100F (JEOL, Akishima, Tokio prefecture, Japan) device. Porosity of the prepared samples was detected by an image analysis using the ImageJ software; for each sample, four individual random scans the average porosity value from which was subsequently calculated were observed.

The microhardness was measured via the Vickers microhardness method with the load of 1 kg and loading time of 10 s using a Zwick-Roell machine (Zwick/Roell—Messphysik KAPPA LA, Zwick/Roell CZ s.r.o., Brno, Czech Republic). The average value for each sample was calculated from a line scan measured through the sintered sample cross-section with 0.5 mm spacing of the individual indentations. As the diameter of the sintered samples was approximately 12 mm (up to ±3 mm), the final average microhardness value was calculated from 13 indents for each sample. The tensile tests were performed on 150 mm long testing samples with circular cross-sections of the diameters of 10 mm at the strain rate of 10^−3^ using a Testometric M500-50CT testing machine (Testometric Co. Ltd., Rochdale, UK). In order to eliminate errors caused by possible inhomogeneities occurring during sintering, four tensile tests were performed for each sintering (and quenching) regime. The reported stress–strain curves in the relevant section then show the ones corresponding the most to the average values for each processing regime.

## 3. Results

### 3.1. Effect of Sintering Temperature on Structures

**Figure 2:**
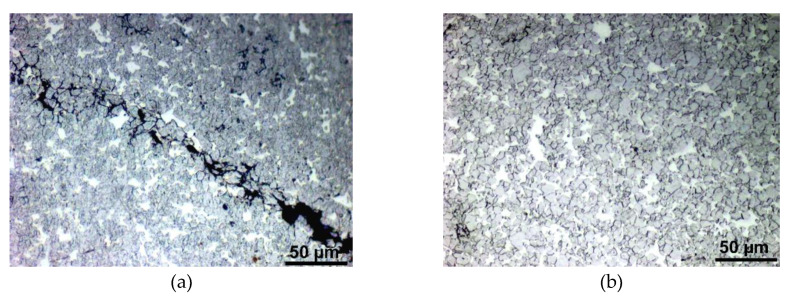

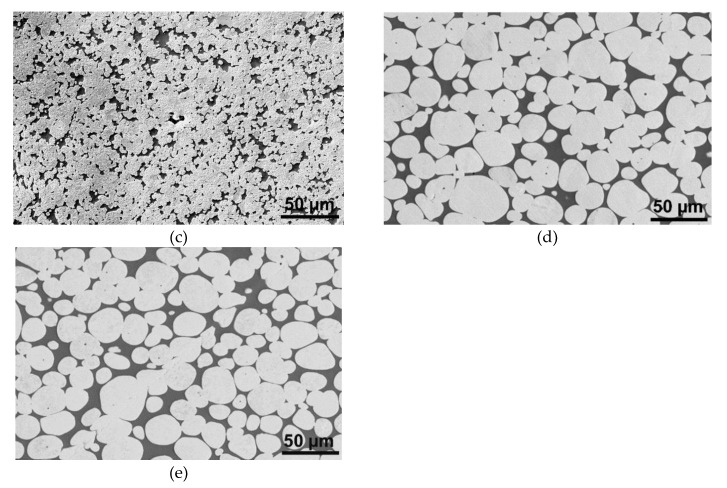
Structure scans of individual sintered pieces: 1450 °C/30 min (**a**); 1475 °C/30 min (**b**); 1500 °C/30 min (**c**); 1525 °C/30 min (**d**); 1550 °C/30 min (**e**). In OM images, white/light areas depict the NiCo matrix, grey areas depict tungsten, and black areas depict voids/cracks.

The results of the analyses investigating the effects of sintering temperature on the structures of the pieces sintered for 30 min revealed significant change in the structure character at the temperature of 1525 °C. Figure 2a shows the optical microscopy structure scan of the sintered-piece prepared at 1450 °C. The scan clearly shows structure inhomogeneity, as well as the presence of voids/gaps; the average porosity for this sample was 4.86%. The optical microscopy scan of structure of the piece sintered at 1475 °C is shown in Figure 2b depicting structure inhomogeneity comparable to the 1450 °C sintered piece. However, the presence of voids is significantly lower when compared to the 1450 °C sample (average porosity of 1.45%). SEM scan of the sample sintered at 1500 °C is shown in Figure 2c. As can be seen, this sintering regime leads to certain structure homogenization and almost complete elimination of porosity, which was as low as 0.23%. As depicted in the SEM scan in Figure 2d, the sintering temperature of 1525 °C results in the formation of the microstructure consisting of tungsten agglomerates and the NiCo matrix. Finally, Figure 2e shows the SEM scan of the structure of the 1550 °C sintered piece, the tungsten agglomerates surrounded by the NiCo matrix in which can clearly be seen, too. Both the 1525 and 1550 °C samples exhibited no residual porosity.

**Figure 3:**
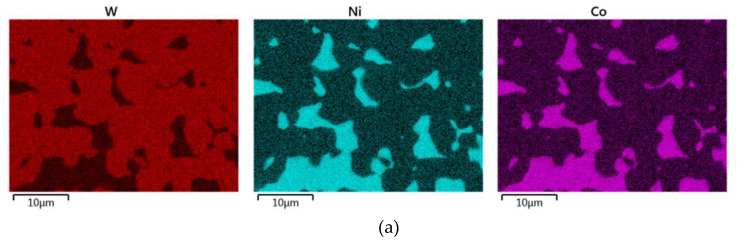

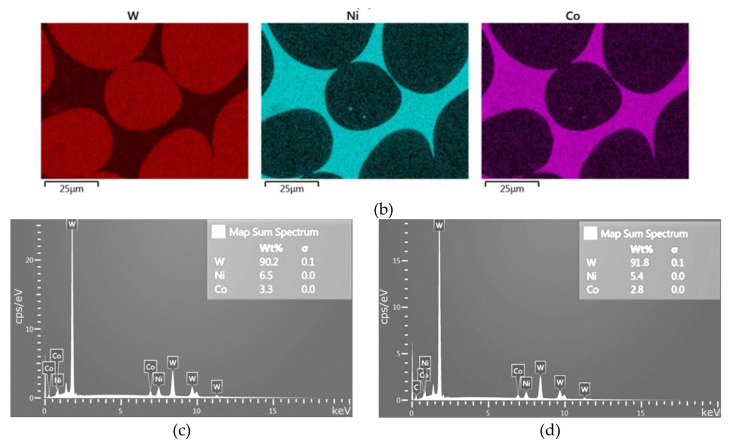
SEM-EDX maps of distribution of individual elements (W-red, Ni-blue, Co-violet) for sintered pieces: 1500 °C/30 min (**a**); 1525 °C/30 min (**b**); quantitative evaluation of individual elements within maps depicted in (**a**) and (**b**) in investigated structures, in wt. %: 1500 °C/30 min (**c**); 1525 °C/30 min (**d**).

In order to examine the effects of the sintering temperature on chemical composition, i.e., distribution of the individual elements within the structures, SEM-EDX analyses were performed for selected sintered pieces. Figure 3a shows the chemical composition for the sample sintered at 1500 °C for 30 min depicted via distributions of the individual elements within the scanned area. Such distribution was typical for the samples sintered at the temperature of 1500 °C and lower. Figure 3c then shows quantitative evaluation of the individual elements within the scanned map. The chemical composition depicted via distributions of the individual elements within the sample sintered at 1525 °C for 30 min is shown in Figure 3b, corresponding quantitative evaluation of the individual elements within the scanned area of the sample is then depicted in Figure 3d. Such distribution of the individual elements was typical for the samples sintered at and above the temperature of 1525 °C. The maps in Figure 3b show that tungsten is primarily concentrated in the agglomerates surrounded by the NiCo matrix.

### 3.2. Effect of Sintering Time on Structures

**Figure 4:**
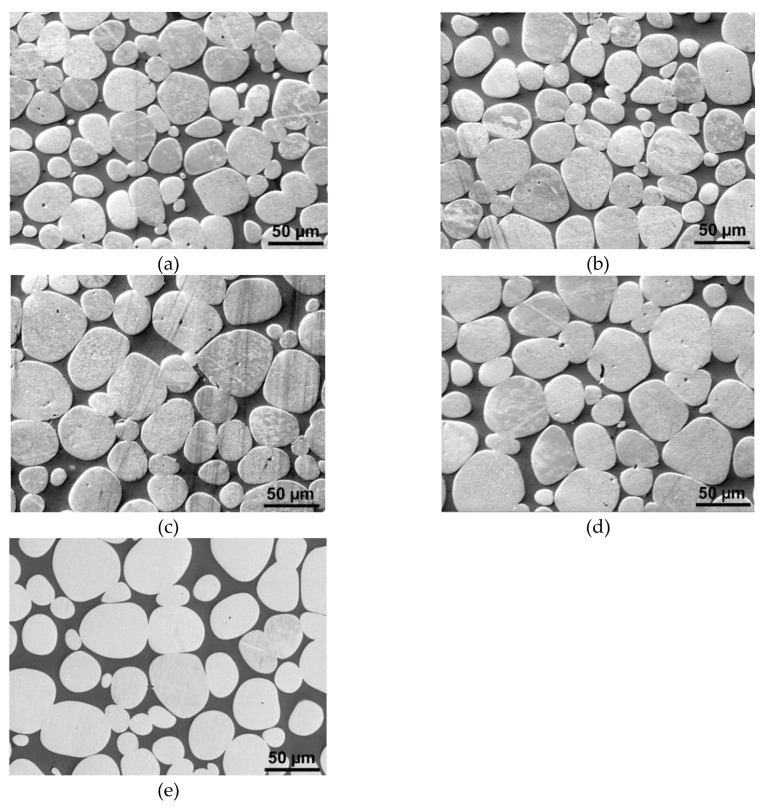
Structure scans of individual pieces sintered at 1500 °C for: 60 min (**a**); 90 min (**b**); 120 min (**c**); 150 min (**d**); 180 min (**e**).

The subsequent research step was the investigation of the effects of variable sintering time at the critical sintering temperature of 1500 °C. The results of the analyses are depicted in Figure 4a–e, the SEM scans of samples sintered for 60, 90, 120, 150, and 180 min in which are depicted, respectively. Evidently, increasing the sintering time at the temperature of 1500 °C to at least 60 min leads to the formation of the microstructures consisting of tungsten agglomerates surrounded by the NiCo matrix.

**Figure 5:**
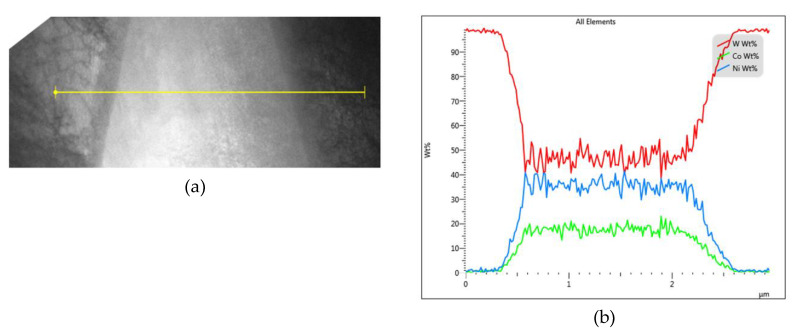
TEM scan showing a W-NiCo-W interface and measured line scan area for sample sintered at 1500 °C for 180 min (**a**); chemical composition of measured line scan (**b**).

Despite the fact that increasing the sintering time has favorable effects on the structure character when examined via SEM, more detailed TEM investigations showed that increasing the time dwell on the high temperature leads to inter-diffusion of tungsten into the NiCo matrix, as documented by Figure 5a,b. Figure 5a shows a TEM scan of a W-NiCo-W interface within the sample sintered at 1500 °C for 180 min, Figure 5b then shows the chemical composition (in wt. %) along a line scan measured across the mentioned interface. As can be seen, the content of tungsten increased to about 50 wt. % in the inter-agglomerate region by the effect of increased sintering time (negligible tungsten content within the NiCo matrix was observed for the sample sintered at 1500 °C for 60 min (not shown here)).

### 3.3. Effect of Quenching on Structures

**Figure 6:**
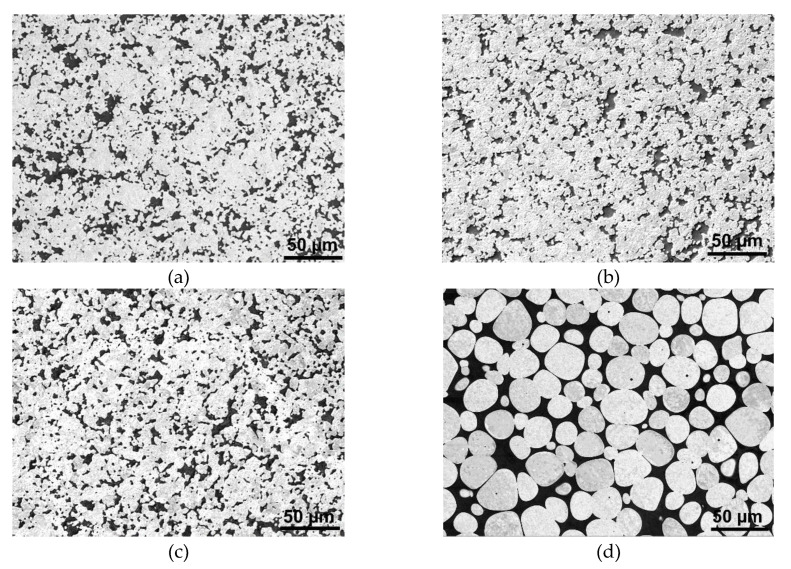

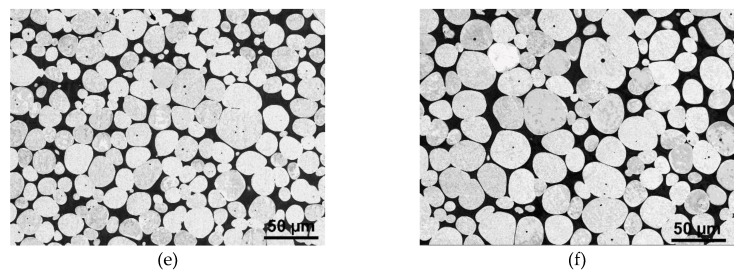
Structure scans of individual quenched pieces sintered at: 1450 °C/30 min (**a**); 1475 °C/30 min (**b**); 1500 °C/30 min (**c**); 1500 °C/180 min (**d**); 1525 °C/30 min (**e**); 1550 °C/30 min (**f**).

Investigation of the effects of quenching into room temperature water right after sintering was performed for all the samples sintered in the temperature range from 1450 to 1550 °C for 30 min, as well as the sample sintered at 1500 °C for 180 min. As shown by the structure analyses the results of which are depicted in Figure 6a–f, quenching does not have any significant effect on the structure character, as the quenched structures were similar to the non-quenched ones; quenched samples sintered at/above the temperature of 1525 °C for 30 min, and the sample sintered at 1500 °C for 180 min, exhibited the structure consisting of W agglomerates and the NiCo matrix, while the other analyzed samples featured more or less randomly distributed W/NiCo locations.

### 3.4. Mechanical Properties

Microhardness was measured for all the sintered pieces in order to analyze the effects of the sintering temperatures on their properties. The results of the measurements are summarized in Table 1. As regards to the samples sintered for 30 min, the highest average microhardness value was recorded for the sample sintered at 1550 °C. However, the highest microhardness value of all was measured for the sample sintered at 1500 °C for 180 min. This finding led to the performance of a supplementary experiment, in which a sample was sintered at the temperature of 1525 °C for 180 min. However, for the elevated temperature, the effect of increasing sintering time is not positive, as the microhardness decreased dramatically when compared to the sample sintered at 1500 °C for 180 min.

**Figure 7:**
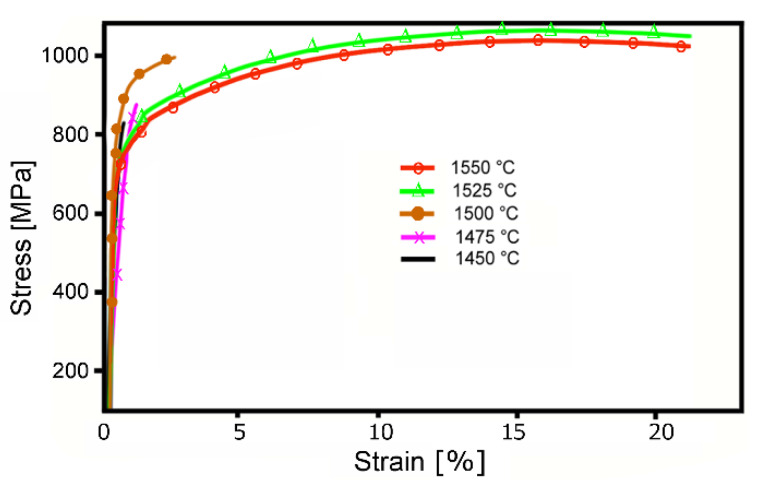
Stress–strain curves for sintered and subsequently quenched pieces.

Last but not least, the tensile tests were performed for the sintered and subsequently quenched pieces; the resulting stress–strain curves are depicted in Figure 7. Ass can be seen from the results, the samples sintered at/below 1500 °C for 30 min, featuring structures with randomly distributed W/NiCo locations and voids, exhibit very low plasticity (lower than 2.5%). The 1450 and 1757 °C samples exhibited the lowest plasticity of less than 1%. On the other hand, structures featuring the homogeneous distribution of tungsten agglomerates within a NiCo matrix exhibit a very high strength of more than 1000 MPa, together with excellent plasticity (elongation to failure exceeding 20%).

## 4. Discussion

Microstructure observations showed that, during sintering at temperatures above the critical value of 1525 °C, the original powder particles consolidated and the structures exhibited the desired distribution of tungsten agglomerates with the sizes of several dozens of micrometres surrounded by a matrix primarily consisting of Ni and Co, whereas the samples sintered below this temperature featured more or less randomly distributed locations with high tungsten or nickel-cobalt contents. Moreover, the structures of pieces sintered below 1525 °C also featured voids and gaps. Sintering at low temperatures thus does not impart the desired structure homogenization and spheroidization of tungsten powder particles into agglomerates. In other words, sintering at temperatures at/above 1525 °C leads to local melting of the alloying elements, the melting temperatures of which are lower than the sintering one (1455 °C for nickel and 1495 °C for cobalt [28]), which supports the development of the desired structure character via diffusion. By this reason, especially the samples sintered below 1500 °C exhibited the presence of voids/gaps, as documented in Figure 2a,b. 

Having performed the first part of the study, we found that the optimum sintering regime among the investigated ones, from the viewpoint of achievement of the required structure character, is the temperature of 1525 °C and time of 30 min. However, as the sintering temperature is definitely higher than the melting temperatures of both the alloying elements, the work pieces exhibit the tendency to gravity sedimentation during sintering, which increases the cross-sectional ovality. In other words, increasing the sintering temperature causes the shape of the cross-section of the sintered pieces to be oval, more than circular. By this reason, an additional experiment with increasing the sintering time at the sintering temperature of 1500 °C was performed. 

Generally, increasing the sintering time was found to affect the mean size of the tungsten agglomerates—it increased with increasing sintering time [29], as well as to support inter-diffusion of the individual elements. Previous neutron powder diffraction measurements identified two individual phases in the sintered pieces featuring the required structure character, the main one of which was α-W featuring the B2 structure (W-B2 phase) [29]. The second phase, NiCo_2_W, with the weight fraction of 6–7% had a pure Ni-like structure (FCC) with the lattice parameter of about 3.60 Å, which is, however, slightly larger than for pure nickel (3.55 Å). This fact indicated alloying of the matrix with larger atoms (atomic radius of W, Ni, and Co is 139, 124, and 125 pm, respectively), and thus revealed the presence of a certain amount of diffused W in the NiCo-based matrix. This phenomenon was explained by Chuvil’deev et al. [30], who documented that introducing distortions to the tungsten lattice, which can be performed by introducing energy via high sintering temperature and/or time, or via subsequent plastic deformation, leads to formation of strong fields of internal stress, which decreases the activation energy necessary for decoupling of tungsten atoms and supports their diffusion to the NiCo matrix. These phenomena are behind the increase in microhardness for the 1500 °C/180 min sintered piece. On the other hand, the 1525 °C/180 min sintered piece exhibited rapid decrease in microhardness when compared to the 1500 °C/180 min one. This can be explained via softening processes occurring within the matrix [17].

The subsequent quenching does not affect the structure character of the sintered pieces. However, it has positive effects on the mechanical properties, testing of which revealed excellent plasticity exceeding 20%, together with the ultimate tensile strength (UTS) of more than 1000 MPa for the pieces sintered at 1525 and 1550 °C for 30 min and quenched. The fact that the UTS of the 1550 °C sintered piece is slightly lower than of the 1525 °C one can be attributed to the occurring restoration phenomena supported by the increased sintering temperature (further temperature increase from 1525 °C introduces similar effects as increasing the sintering time at 1525 °C) [17,28].

## 5. Conclusions

The study focused on the investigations of the effects of sintering temperature, sintering time, and optional subsequent quenching in water on the structures and mechanical properties of a 93W6Ni1Co tungsten heavy alloy. The results revealed the following: 

- 30-min-sintering at temperatures below 1525 °C does not provide sufficient homogenization as the structures feature more or less random locations with high W or NiCo concentrations, as well as voids and gaps. The microhardness is also low for such samples; 

- sintering at or above 1525 °C leads to formation of homogeneous structures featuring W agglomerates surrounded by the NiCo matrix, which also increases the mechanical properties;

- sintering at 1500 °C for 30 min leads to inhomogeneous structure, but increasing the time to at least 60 min results in comparable effects as increasing the sintering temperature, however, increasing the sintering time provokes diffusion of W into the matrix which, on the other hand, increases the microhardness;

- implementation of quenching results in excellent mechanical properties of the quenched pieces sintered at/above 1525 °C—plasticity of almost 20% and strength of more than 1000 MPa.

The optimized preparation regime providing the 93W6Ni1Co alloy with structure and properties being the most favorable for subsequent processing is sintering at 1525 °C for 30 min and subsequent quenching in water.

## Figures and Tables

**Table 1:** Average microhardness for sintered pieces.

Sample Sintering Regime	Average Microhardness [HV]	Standard Deviation
1450 °C/30 min	378.54	14.86
1475 °C/30 min	389.75	13.40
1500 °C/30 min	376.61	10.54
1500 °C/180 min	483.99	5.16
1525 °C/30 min	377.97	9.67
1525 °C/180 min	337.56	7.64
1550 °C/30 min	392.83	11.42
